# Favipiravir for the treatment of patients with COVID-19: a systematic review and meta-analysis

**DOI:** 10.1186/s12879-021-06164-x

**Published:** 2021-05-27

**Authors:** Toshie Manabe, Dan Kambayashi, Hiroyasu Akatsu, Koichiro Kudo

**Affiliations:** 1 Nagoya City University Graduate School of Medicine, 1 Kawasumi, Mizuho-cho, Mizuho-ku, Nagoya City, Aichi 467-8601 Japan; 2 Nagoya City University West Medical Center, Nagoya City, Aichi Japan; 3 Showa Pharmaceutical University, Tokyo, Japan; 4 Yurin Hospital, Tokyo, Japan; 5 Waseda University Regional and Inter-Regional Organization, Tokyo, Japan

**Keywords:** Favipiravir, COVID-19, SARS-CoV-2, Viral clearance, Clinical improvement

## Abstract

**Background:**

Favipiravir possesses high utility for treating patients with COVID-19. However, research examining the efficacy and safety of favipiravir for patients with COVID-19 is limited.

**Methods:**

We conducted a systematic review of published studies reporting the efficacy of favipiravir against COVID-19. Two investigators independently searched PubMed, the Cochrane Database of Systematic Reviews, MedRxiv, and ClinicalTrials.gov (inception to September 2020) to identify eligible studies. A meta-analysis was performed to measure viral clearance and clinical improvement as the primary outcomes.

**Results:**

Among 11 eligible studies, 5 included a comparator group. Comparing to the comparator group, the favipiravir group exhibited significantly better viral clearance on day 7 after the initiation of treatment (odds ratio [OR] = 2.49, 95% confidence interval [CI] = 1.19–5.22), whereas no difference was noted on day 14 (OR = 2.19, 95% CI = 0.69–6.95). Although clinical improvement was significantly better in the favipiravir group on both days 7 and 14, the improvement was better on day 14 (OR = 3.03, 95% CI = 1.17–7.80) than on day 7 (OR = 1.60, 95% CI = 1.03–2.49). The estimated proportions of patients with viral clearance in the favipiravir arm on days 7 and 14 were 65.42 and 88.9%, respectively, versus 43.42 and 78.79%, respectively, in the comparator group. The estimated proportions of patients with clinical improvement on days 7 and 14 in the favipiravir group were 54.33 and 84.63%, respectively, compared with 34.40 and 65.77%, respectively, in the comparator group.

**Conclusions:**

Favipiravir induces viral clearance by 7 days and contributes to clinical improvement within 14 days. The results indicated that favipiravir has strong possibility for treating COVID-19, especially in patients with mild-to-moderate illness. Additional well-designed studies, including examinations of the dose and duration of treatment, are crucial for reaching definitive conclusions.

**Supplementary Information:**

The online version contains supplementary material available at 10.1186/s12879-021-06164-x.

## Background

Severe acute respiratory syndrome coronavirus 2 (SARS-CoV-2) was first identified in Wuhan, Hubei Province, China [1], and it is the causative agent of the coronavirus disease 2019 (COVID-19) pandemic [2]. By the end of September, 2020, it brings the cumulative numbers to over 61.8 million cases and 1.4 million death globally since the start of the pandemic [3]. COVID-19 is caused by the novel virus, so that pathophysiology and effective treatment methods were unknown, and vaccine was not available at the first stage of the outbreak. Although the discovery of effective treatment methods were urgently required, the discovery of new antiviral agents against SARS-CoV-2 takes a long time and it is not straightforward task.

SARS CoV-2 is a positive strand RNA (+RNA) virus, and is a member of the coronaviridae family. SARS CoV-2 is a single-stranded RNA beta-coronavirus encoding an RNA-dependent RNA polymerase (RdRp) and proteases. Both RdRp and viral proteases are considered important targets for potential therapeutic agents. Favipiravir, previously known as T-705, is a prodrug of the purine nucleotide favipiravir ribofuranosyl-5′-triphosphate [4]. The active agent inhibits RNA polymerase, halting viral replication [4]. Favipiravir was approved in 2014 by the Japan Pharmaceuticals and Medical Devices Agency under the brand name AVIGAN® for the treatment of novel and re-emerging influenza virus infection [5]. Several studies described its effectiveness against other RNA viruses such as Ebola virus [6], as well as the effectiveness against rhinovirus and respiratory syncytial virus [7]. In vitro, the 50% effective concentration (EC_50_) of favipiravir against SARS-CoV-2 was 61.88 μM/L in Vero E6 cells [8]. Thus, favipiravir possess high potential for treating patients with COVID-19. However, research examining the efficacy and safety of favipiravir in patients with COVID-19 is limited.

The aim of the present study was to review systematically on the application of favipiravir for patients with COVID-19 to identify empirical evidence of its efficacy.

## Methods

This systematic review and meta-analysis was conducted in accordance with the Preferred Reporting Items for Systematic Reviews and Meta-Analyses (PRISMA) statement and the statement by the Meta-analysis of Observational Studies in Epidemiology (MOOSE) group [9, 10].

### Eligibility criteria and outcome measures

Studies fulfilling the following selection criteria were included in the meta-analysis: (1) study design and language: randomized clinical trials (RCTs), observational studies, and case series involving > 10 patients written in the English language; (2) population: patients with laboratory-confirmed SARS-CoV-2 infection who were hospitalized or treated in clinics; (3) intervention: administration of favipiravir; (4) comparison intervention: placebo, standard of care (SOC), remdesivir, lopinavir/ritonavir, other available antivirals, hydroxychloroquine (HQ), different dosages of favipiravir, combination therapy with favipiravir, or no comparator; (5) primary outcomes: viral clearance and clinical improvement including improvement of chest computer tomography (CT); and (6) secondary outcomes, any outcome variable. The exclusion criteria were as follows: (1) ≤10 patients in case series, (2) no reporting of outcome variables, and (3) insufficient or incomplete data.

### Information sources and search strategy

Two investigators (T.M. and D.K.) independently searched for eligible studies in PubMed, the Cochrane Library, and MedRxiv from inception to September 12,020. We used the following key words: “novel coronavirus” OR “new coronavirus” OR “emerging coronavirus” OR “2019-nCoV” OR “COVID-19” OR “SARS-CoV-2” AND “favipiravir” OR “avigan” OR “T-705.” We searched the reference lists of all included studies, reviews, and clinical trial registries for ongoing trials investigating the efficacy or safety of favipiravir for patients with COVID-19. We also reviewed the reference lists of eligible studies using Google Scholar and performed a manual search to ensure that all appropriate studies were included.

### Data extraction

Two reviewers (T.M. and D.K.) extracted the data independently. Articles retrieved in the search were stored in a citation manager (EndNote X9; Thomson Reuters, New York, NY, USA). After removing redundant articles, titles, abstracts, and then full-text articles were investigated. We extracted the following data: study design, observational period, study site, and inclusion/exclusion criteria of each study. Outcome variables were extracted into predesigned data collection forms. We verified the accuracy of data by comparing the collection forms of each investigator. Any discrepancies were resolved through discussion among the authors.

### Risk of bias assessment

For clinical trials and before and after controlled trials, we assessed the risk of bias (“low risk,” “some concerns,” or “high risk”) in the overall effect of favipiravir on viral clearance and clinical improvements using version 2 of the Cochrane Risk of Bias Assessment Tool [11]. Risk of bias assessments were performed independently by two investigators (T.M. and D.K.), with disagreements resolved through discussion. We used the Grading of Recommendations Assessment and Evaluation approach [12] to assess the certainty of the evidence that favipiravir reduced the time to viral clearance and contributed to clinical improvement.

### Data analysis

Throughout the meta-analysis, we estimated the odds ratios (ORs) or the proportions of patients for primary outcome variables with 95% confidence intervals (CIs) using a random-effects model (generic inverse variance method). To assess the proportions of the outcome variables among patients with COVID-19, the standard error was calculated using the Agresti–Coull method [13]. Heterogeneity among the original studies was evaluated using the *I*^2^ statistic [14]. Publication bias was examined using a funnel plot. For all analyses, significance levels were two-tailed, and *p* < 0.05 was considered significant. All statistical tests were performed using Review Manager (RevMan) ver. 5.3.5 (Cochrane Collaboration, Copenhagen, Denmark) [15].

## Results

### Study selection and characteristics

**Fig. 1:**
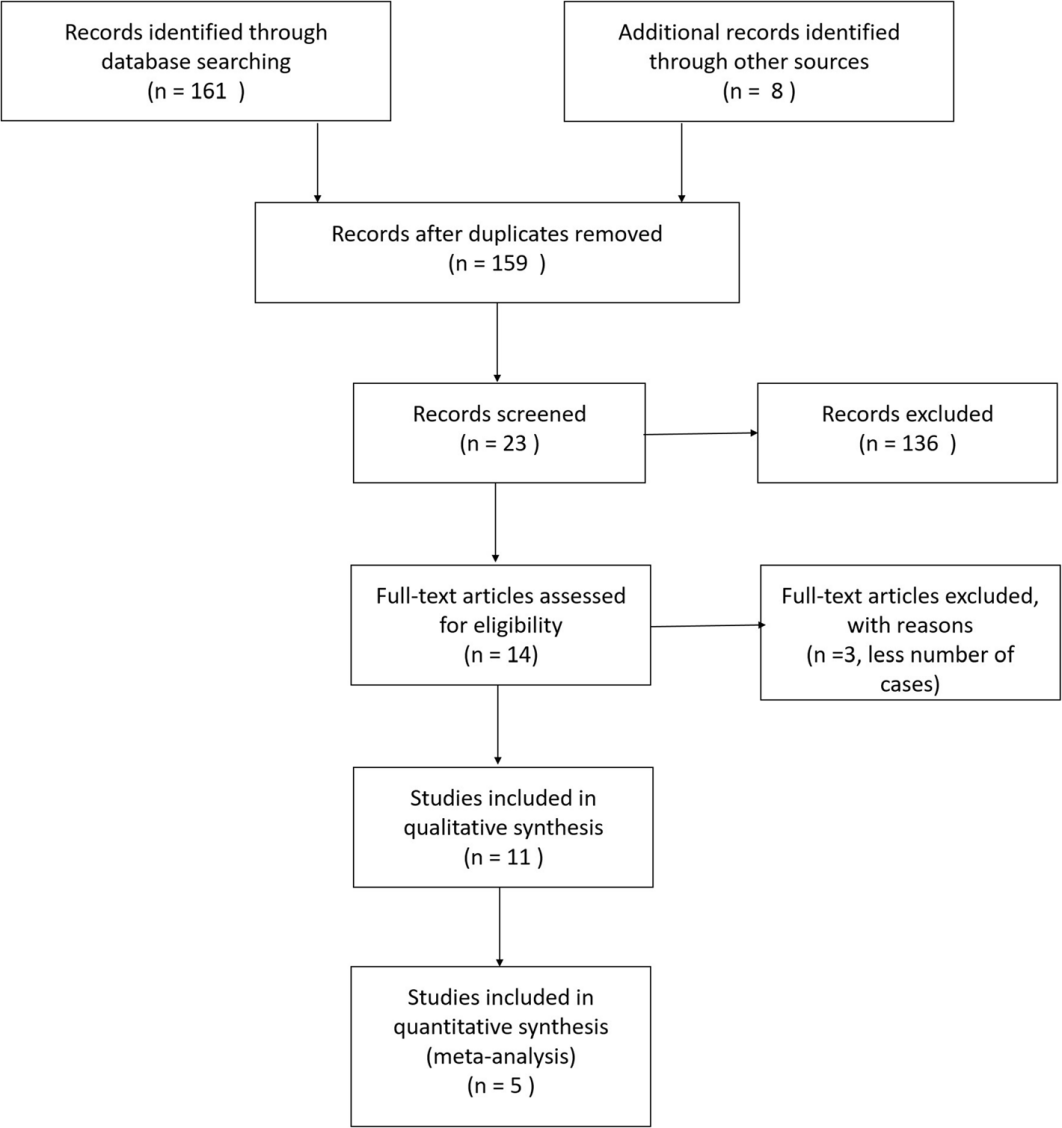
PRISMA flow diagram. N is the number of articles

Of the 163 references screened, 11 studies were eligible (Fig. 1).

Table 1 presents the characteristics of the included studies.

**Table 1:** Backgrounds of patients with COVID-19 among the eligible studies

Study, year (Country)	Observational period	Intervention	Comparator	Dose of favipiravir 1. First day 2. Following days	Duration of favipiravir	No. of participants	Sex-Male, n (%)	Age, median (IQR), years	Severity of COVID-19, n (%)	Times between onset to the initiation of treatment
**Randomized-controlled study**										
Chen C, 2020 [16] (China)	Feb. 20 to Mar. 1, 2020	Favipiravir	Umifenovir	1. 3200 mg 2. 1200 mg	10 days	236	110 (46.6)	≥ 65, n (%) 70 (29.7)	Moderate, 88.6% Severe 10.2% Critical, 1.3%	–
Lou Y, 2020 [17] (China)	Feb 3. -	Favipiravir	Baloxavir marboxil or Control	1. 1600 mg or 2200 mg 2. 1800 mg	14 days	29	21 (72.4)	Mean (SD), 52.5 (12.5)	–	–
Ivashchenko AA, 2020 [18] (Russian Federation)	April and May 2020	Favipiravir	SOC	1. 3200 mg 2. 1200 mg or 1. 3600 mg 2. 1600 mg	14 days	60	–	aged 60 and older	Moderate COVID-19 pneumonia	–
Doi Y, 2020 [19] (Japan)	March 2–May 18, 2020	Favipiravir	Early (day 1) vs. Late (day 6) treatments	1. 3200 mg 2. 1600 mg	Up to 19 dose over	69	54 (61.4)	50 (38–64.5)	Asymptomatic or mild	–
**Before-after nonrandomized controlled study**										
Cai Q, 2020 [20] (China)	Jan. 30 - Feb. 14, 2020	Favipiravir	Lopinavir/ritonavir	1. 3200 mg 2. 1200 mg	14 days	80	35 (43.8)	47 (35.75–61)	Moderate	–
**Observational study or Case series**										
Rattanaumpawan P, 2020 [21] (Thailand)	Jan. 1 to April 30, 2020	Favipiravir	Clinical improvement on day 7	examining a loading dose		274	39 (61.9)	48 (22–85)	NEWS2 score-median (IQR), 4 (4–5)	Median, 8 (0–28) days
Yamamura H, 2020 [22] (Japan)	April 2–27, 2020	Favipiravir & methylprednisolone^b^	–	1. 3600 mg 2. 1600 mg	14 days	13	9 (69)	Mean (SD), 63 (12)	Severe requiring MV	8.7 days (range, 4–13)
Doi K, 2020 [23] (Japan)	April 6–21, 2020	Favipiravir & nafamostat mesylate	–	1. 3600 mg 2. 1600 mg	14 days	11	10 (91)	68 (60–69)	Admitted to ICU	–
Murohashi K, 2020 [24] (Japan)	NA	Favipiravir & methylprednisolone^c^	–	1. 3600 mg 2. 1600 mg	14 days	11	8 (72.7)	70 (45–82)	severe and required oxygen administration or SpO_2_ ≤ 93%	to admission, 6.4 days^a^
Çalik BaŞaran N, et al. [25] (Turkey)	March 20–April 30, 2020	Favipiravir	HQ only, HQ plus AZ	–	–	174	91 (52.3)	45.5 (range, 19–92) ≥65 years, n (%), 24 (13.8)	Mild, 20.1% Moderate, 61.5% Severe, 18.4%	to admission, 3 days (0–14) ^a^
Yaylaci S, et al. [26] (Turkey)	March 25 – May 5, 2020	Favipiravir	–	–	at least 5 days	62	36 (58.1)	Median (range), 64 (37–89)	Mild or moderate, 72.6%; Severe, 27.4%	–

During the study, patients in all groups were allowed to use pathogenetic and symptomatic treatment. Patients in the favipiravir groups were not allowed to use other antiviral or antimalarial drugs

*IQR* inter quartile range, *SOC* standard of care, *MV* mechanical ventilation; ^a^ time from onset to the admission

^b^ Methylprednisolone (1000 mg for 3 days) begun from 5th day from the initiation of favipiravir

^c^ Methylprednisolone (80 mg/day for 3 days), followed by 250 mg/day for 3 days

*COVID-19* coronavirus disease 2019, *SpO*_*2*_ blood oxygen saturation, *HQ* hydroxychloroquine, *AZ* azithromycin, *SD* standard deviation, *IQR* interquartile range, *NEWS2* National Early Warning Score, *NA* not available

Among the 11 studies, three studies were RCTs [16, 18–20], one study was a non-randomized controlled study [17], one study was a before and after nonrandomized controlled study [21], and six studies were observational studies or case series [22–26]. Among the comparative studies, the comparators included umifenovir [17], baloxavir marboxil [18], standard of care (SOC) [19], lopinavir/ritonavir [21], and HQ alone or in combination with azithromycin [25]. An RCT that examined the early or late initiation of favipiravir treatment was the only study investigating asymptomatic or mildly ill patients [19]. Although the sample sizes were small, two case series assessed combination therapy involving favipiravir plus nafamostat mesylate [23] or methylprednisolone [22, 24] for patients with severe COVID-19.

The dose of favipiravir generally matched the standard dose for treating influenza infection, namely 1600 mg twice daily on the first day followed by 600 mg twice daily, but in some eligible studies, the dose was 1800 mg twice daily on the first day followed by 800 mg twice daily. One study examined the loading dose [22]. Among the 11 eligible studies, the duration of favipiravir therapy was primarily 14 days [18, 19, 21, 23–25].

### Assessment of bias in studies comparing favipiravir with other antivirals or standard of care among patients with COVID-19

Data on viral clearance and clinical improvement were available for four trials [16–19] and the before and after controlled study [20]. We identified a high risk of bias, and the evidence was assessed at “low” for viral clearance and clinical improvement (Figs. S1–S2).

The major qualitative outcomes of each study are presented in Table 2.

**Table 2:** Outcomes for patients with COVID-19 among eligible studies

	Outcome variables		
	time to viral clearance	time to symptom resolution	Others
Chen C [16]	–	Clinical recovery rate of Day 7: Favipiravir: Total, 61.21% Moderate, 71.43% Severe or critical, 5.56% those in Arbidol: Total, 51.67% Moderate, 55.86% Severe or critical, 0	No mortality both in favipiravir and arbidol groups
Lou Y [17]	Viral clearance in day 7 Favipiravir, 44%, Baloxavir, 60% Control, 50% Viral clearance in day 14 Favipiravir, 77%, Baloxavir, 70% Control, 100%	Median days (IQR) to clinical improvement: Favipiravir, 14 (6–38) Baloxavir, 14 (6–49) Control, 15 (6–24)	–
Ivashchenko [18]	Viral clearance on day 5: Favipiravir, 62.5% SOC group, 30.0% Viral clearance on day 10: Favipiravir, 92.5% SOC group, 80.0%	Chest CT improvement by day 15 Favipiravir, 90% SOC group, 80% . Median days to body temperature normalization (< 37oC) were 2 days (IQR 1–3) in favipiravir group, while those in SOC group were 4 days (IQR 1–8).	Two patients on Favipiravir 1600/600 mg were moved to intensive care unit, received mechanical ventilation and later died.
Doi Y [19]	Viral clearance within 6 days were the Early treatments, 66.7% Late treatments, 56.1% Median days to viral clearance were Early treatments, 12.8 days Late treatments, 17.8 days	Median days to hospital discharge: Early treatments, 14.5 days Late treatments, 20.0 days Time to relieve from high fever (≥37.5 °C) Early treatments, 2.1 days Late treatments, 3.2 days	–
Cai Q [20]	Median (IQR) days to viral clearance: Favipiravir, 4 day (2.5–9) Comparators, 11 (8–13) days	Chest CT improvement on day 9 were 56.3% in favipiravir group, and 35.6% in the comparator group. Chest CT improvement on day 14 were 91.4% in favipiravir group, and 62.2% in the comparator group.	Logistic regression of changes in lung CT: antiviral therapy (OR, 3.190), and fever (OR, 3.622). Cox regression of viral clearance T lymphocyte count (HR, 1.002); Time form onset to treatment (HR, 1.217); FPV vs. LPV/RTV (HR, 3.434)
Rattanaumpawan [21]	–	Clinical improvement for Day 7: All patients, 42 (66.7%) Clinical improvement for Day 14: All patients, 54 (85.7%) Clinical improvement for Day 28: All patients, 57 (90.5%)	Day 14, 28 mortality rate, n (%) all patients: 1 (1.6), 3 (4.8) Required MV or ECMO, n (%) Before favipiravir, 4 (6.3) After favipiravir, 4 (6.3) Duration of therapy, median (range), Multivariate analysis revealed three poor prognostic factors for Day-7 clinical improvement [odds ratio (95%CI); *p*-value]: older age [0.94 (0.89–0.99); *p* = 0.04], higher baseline NEWS2 score [0.64 (0.47–0.88); *p* = 0.006], and lower favipiravir loading dose (≤45 mg/kg/day) [0.04 (0.005–0.4); *p* = 0.006].
	–	Duration of therapy, median (range), days: clinical improvement on Day 7, 11.5 (2–16) No clinical improvement on day 7, 12 (2–17)	
Yamamura H [22]	–	–	Died 1 patient due to DIC. P/F changed very little over the first 6 days and then gradually recovered. IL-6 peaked on day 4 and decreased thereafter. Presepsin peaked on day 3, remained about the same until 6, and then decreased.
Doi K [23]	–	–	1 patient who had a do-not-resuscitate order, died on ICU day 7. No interruption of antiviral treatment occurred due to adverse drug reactions except for one patient who developed hyperkalemia on day 9 (by nafamostat mesylate). All 11 patients had at least 33 days of hospital follow-up. Seven patients were successfully weaned from MV [median duration of MV 16 days (IQR, 10 to 19 days)] and 9 and 7 patients were discharged from the ICU and the hospital, respectively
Murohashi K [24]	The mean time to first-time negative conversion of viral RNA was 18 days in 6 confirmed negative cases.		4 patients discharged; 6 patients have no oxygen at rest; 1 patient worsended on the day of admission, and was transferred to another hospital for ventilator management.
Çalik BaŞaran [25]	–	Median (range) days to clinical improvement: HQ alone, 1 (1–6) HQ and AZ, 1.5 (1–11) Favipiravir, 6 (1–20) Median days of defervescence: HQ alone, 1 (0–4) HQ and AZ, 1 (0–11) Favipiravir, 3 (0–8)	8.5% of patients were transferred to the ICU, 2.2% of patients died. Median (range) length of hospital stay, were 2 (1–21) in HQ alone, 4 (1–15) in HQ and AZ, and 7.5 (2–24) days in favipiravir groups.
Yaylaci S [26]	–	–	Among the examined hemarological parameters before and after favipiravir, RBC, hemoglobin level, hematocrit level, neutrophil count were decreased, and lymphocyte count, platelet count were increased with the statistical significance.

*COVID-19* coronavirus disease 2019, *SOC* standard of care, *CT* computed tomography, *IQR* interquartile range, *OR* odds ratio, *HR* hazard ratio, *DIC* disseminated intravascular coagulation, *ICU* intensive care unit, *MV* mechanical ventilation, *ECMO* extracorporeal membrane oxygenation, *NEWS2* National Early Warning Score, *HQ* hydroxychloroquine, *AZ* azithromycin, *RBC* red blood cell, *FPV* favipiravir, *LPV* lopinavir, *RTV* ritonavir, *CI* confidence interval, *IL-6* interleukin-6

**Fig. 2:**
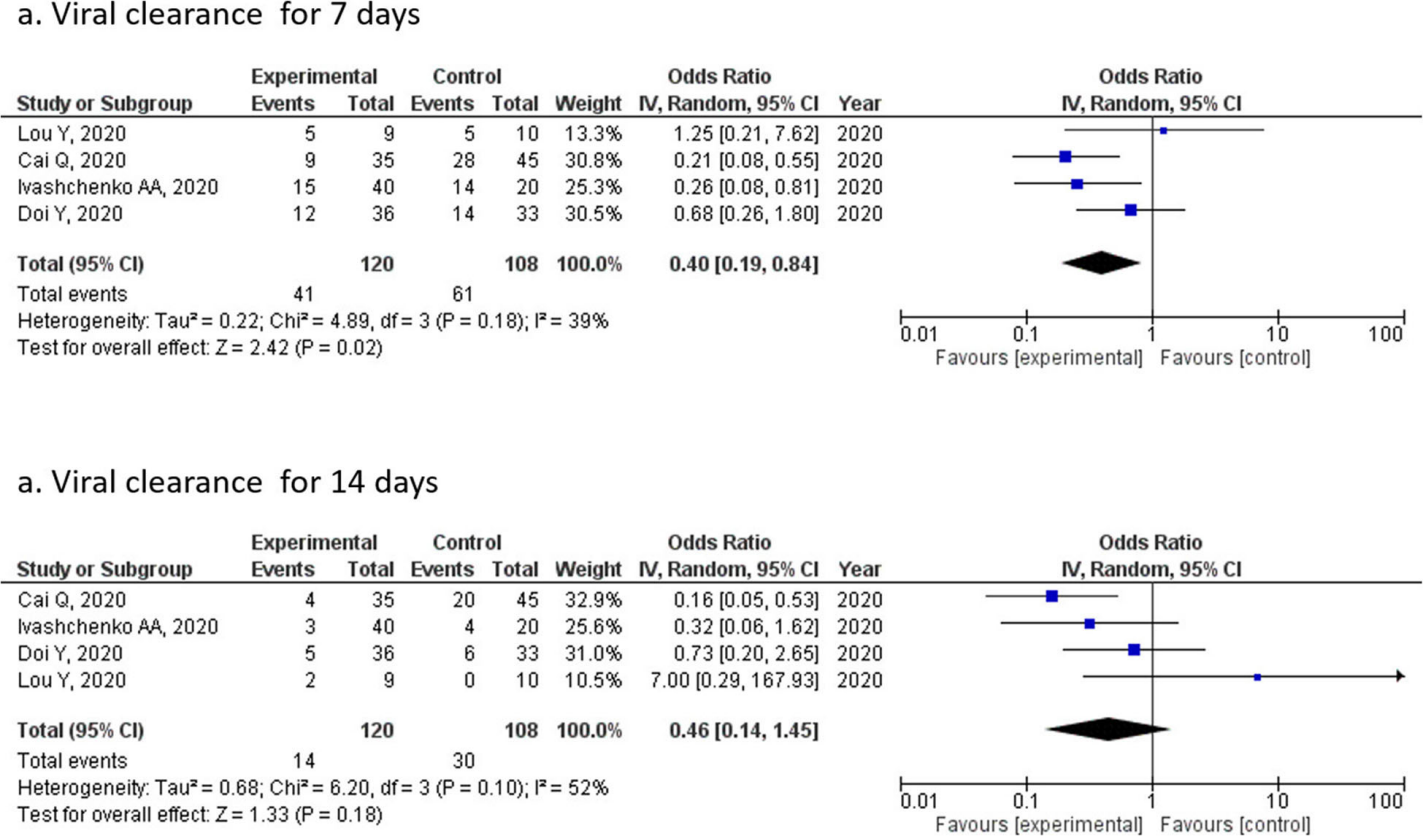
Forrest plots for viral clearance for patients with COVID-19 who were treated with favipiravir or a comparator for (**a**) 7 or (**b**) 14 days

Of the five trials with data on the primary outcomes, we estimated the ORs for the association between favipiravir treatment and viral clearance using meta-analysis (Fig. 2). The OR of viral clearance on day 7 was 0.40 (95% CI = 0.19–0.84) with statistical difference (*p* = 0.02) (Fig. 2a), whereas that on day 14 was 0.46 (95% CI = 0.14–1.45), with no significant difference (*p* = 0.18) (Fig. 2b) noted between the two points.

**Fig. 3:**
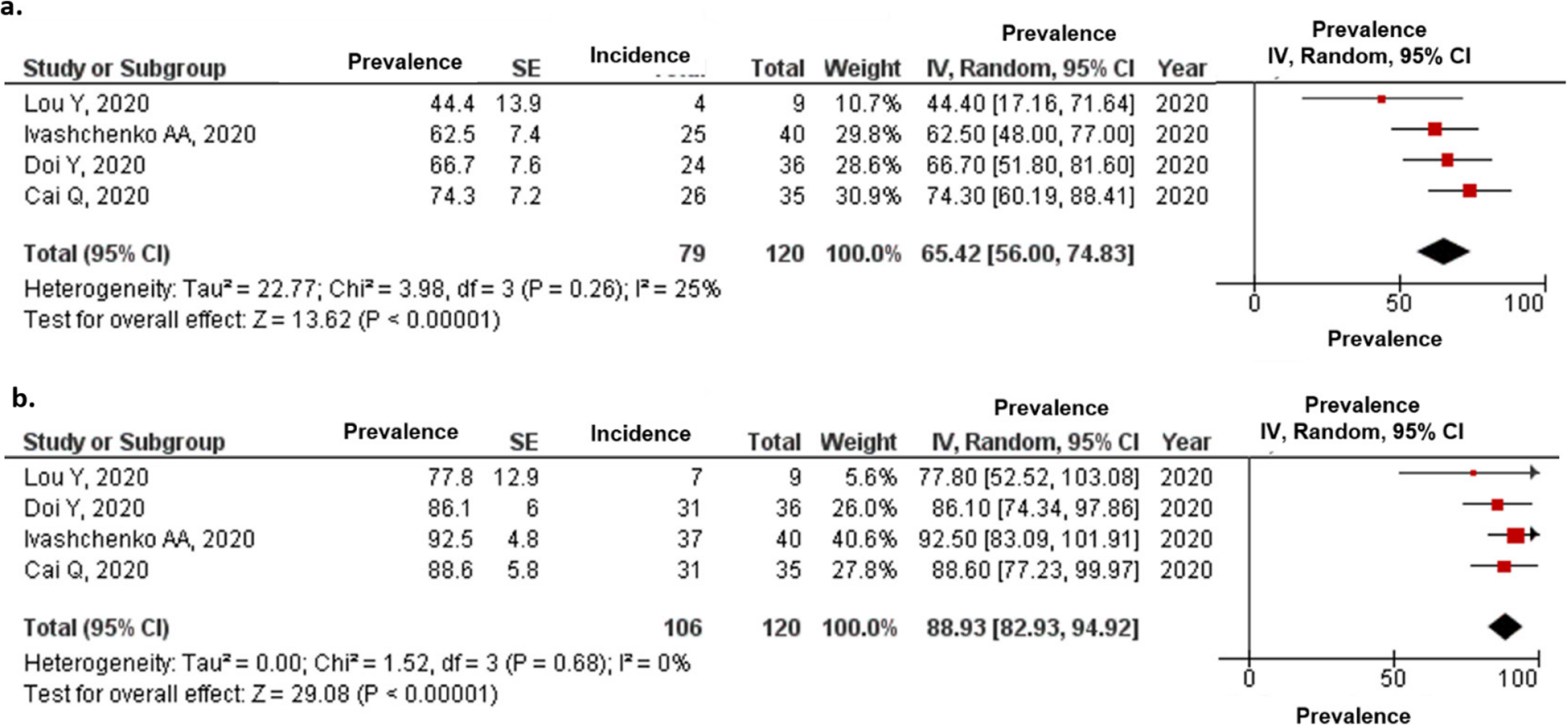
Proportions of patients with COVID-19 who achieved viral clearance on (**a**) 7 and (**b**) 14 days from the initiation of treatment

We also estimated the proportion of patients with viral clearance in the favipiravir (Fig. 3) and comparative treatment arms (Fig. S3).

The estimated proportions of patients with COVID-19 who achieved viral clearance in the favipiravir arm on days 7 and 14 were 65.42 and 88.9% (Fig. 3), respectively, compared with 43.42 and 78.79%, respectively, for the comparator arm (Fig. S3).

### Clinical improvement with favipiravir versus other antivirals or standard of care among patients with COVID-19

**Fig. 4:**
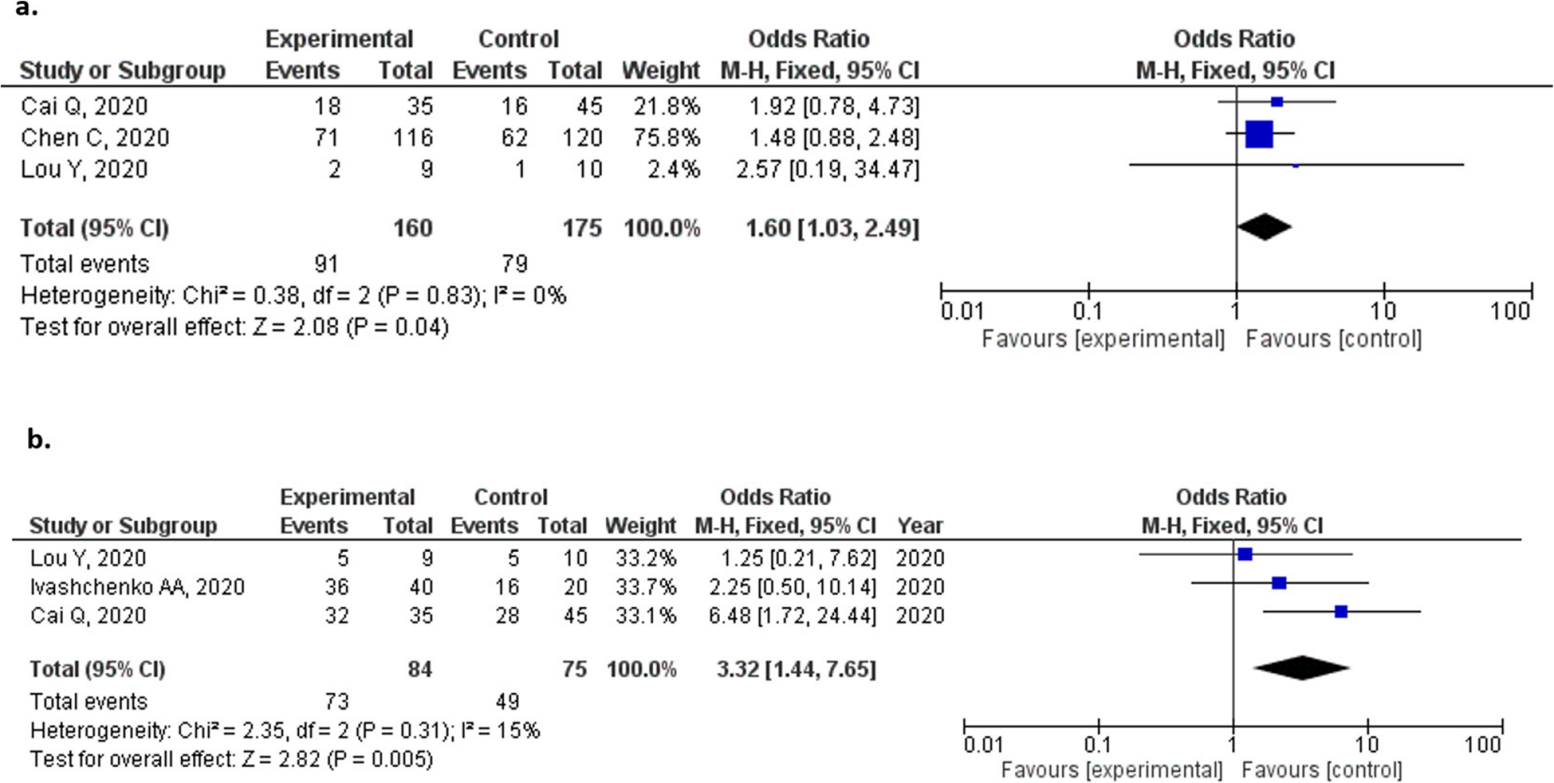
Forrest plots of clinical improvement for patients with COVID-19 treated with favipiravir for (**a**) 7 and (**b**) 14 days

In terms of clinical improvement, the definition of clinical improvement varied among the studies as follows: continuous (> 72 h of recovery of body temperature, respiration rate, oxygen saturation and cough relief after treatment [16]); improvements on the seven-category ordinal scale, which references the National Early Warning Score or live discharge from the hospital, whichever came first [17]; and chest CT improvement [18, 20]. Despite the various definitions, favipiravir was associated with better clinical improvement than comparative therapy after 7 (OR = 1.60, 95% CI = 1.03–2.40, *p* = 0.04; Fig. 4a) and 14 days of treatment (OR = 3.03. 95% CI = 1.17–7.80, *p* = 0.02; Fig. 4b).

**Fig. 5:**
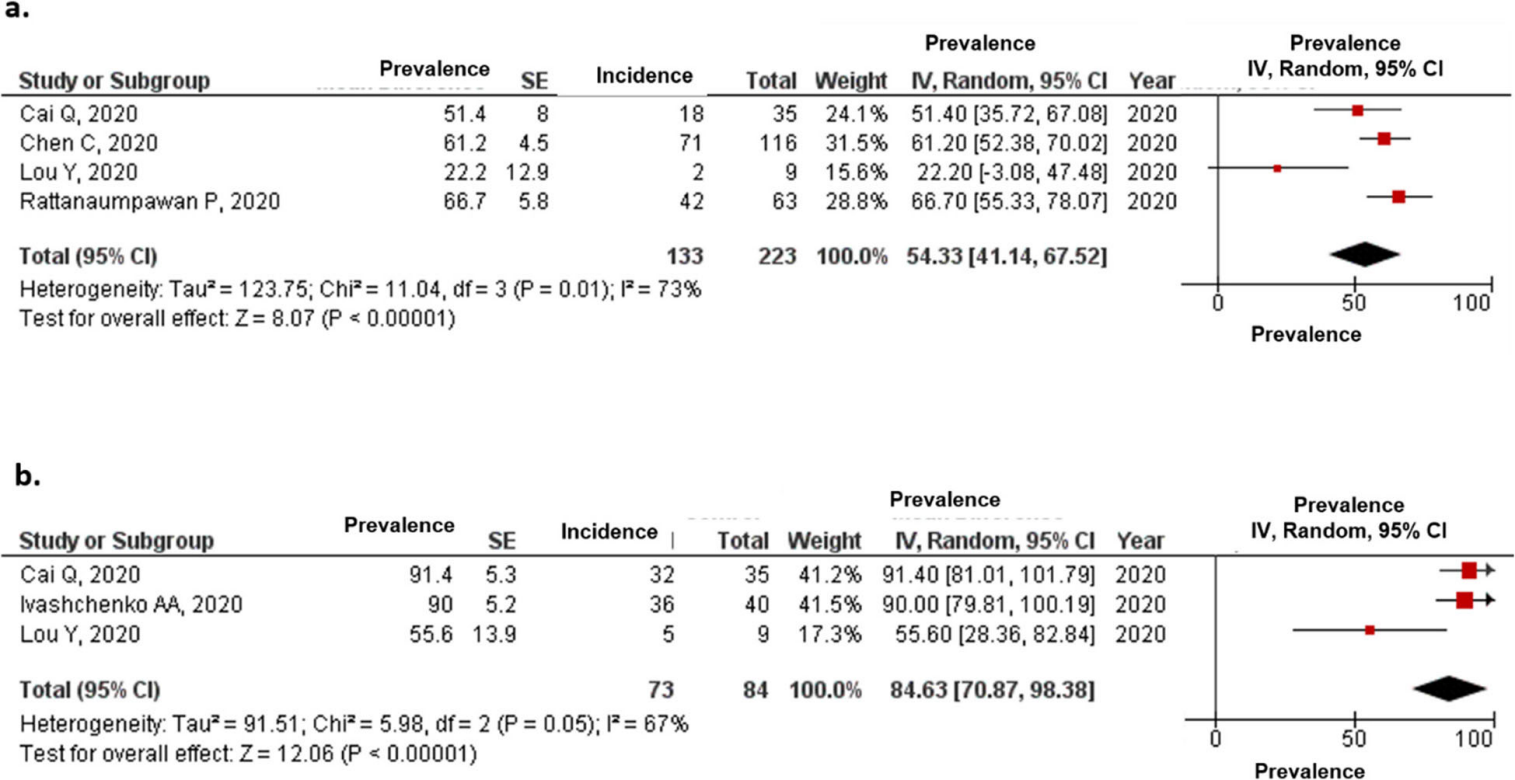
Proportions of patients with COVID-19 who had clinical improvement until (**a**) 7 and (**b**) 14 days from the initiation of treatment

The estimated proportions of patients with clinical improvement in the favipiravir group after 7 and 14 days were 54.33 and 84.63%, respectively (Fig. 5), compared with 34.40 and 65.77%, respectively, for the comparator group (Fig. S4).

### Secondary outcomes

Table 2 presents the various outcomes reported in each study. Among the comparative studies of favipiravir, most patients had moderate COVID-19, and death was observed in only two patients in the favipiravir group who had underlying diseases, such as diabetes mellitus, artificial hypertension, and obesity [18]. In two observational studies that examined the combination of favipiravir plus nafamostat mesylate [23] or methylprednisolone [24] in patients with severe COVID-19 reported one death each. The morality rates of the observational study that examined the loading dosage of favipiravir for patients including severe disease were 1.6% on day 14 and 4.8% on day 28 [22]. The median times to body temperature normalization in the favipiravir and standard of care arms in one study were 2 and 4 days, respectively [18], and those in patients with early and late favipiravir initiation were 2.1 and 3.2 days, respectively [19]. A study comparing HQ alone, HQ plus azithromycin, and favipiravir-containing regimens found that the length of hospital stay was shortest in the HQ alone group [25].

### Adverse reactions of favipiravir

The reported adverse reactions of favipiravir in each study are presented in Table 3.

**Table 3:** Adverse reactions of favipiravir

	Reported adverse drug reactions	
	Favipiravir	Comparator drug
Chen C, et al. (China)	Antiviral-associated adverse effects among favipiravir group were (n, %): Abnormal LFT, 10 (8.62); Raised serum uric acid, 16 (13.79); Psychiatric symptoms reactions, 5 (4.31); Digestive tract reactions, 16 (13.79)	Among arbidol group (n, %): Abnormal LFT, 12 (10.00); Raised serum uric acid, 3 (2.50); Psychiatric symptoms reactions, 1 (0.83); Digestive tract reactions, 14 (11.67)
Lou Y, et al.	Respiratory failure occurred in 14 patients. Other adverse events were generally mild or moderate among the three Groups. The most frequent adverse events 2 occurring in the study population were similar among all groups, including elevation of 3 triglyceride (20 events), liver function abnormality (18 events), rash (7 events), and diarrhea (4 events). No abnormal 5 serum creatinine was found in all patients.	
Ivashchenko AA, et al. (Russian Federation)	Adverse drug reactions were reported in 7/40 (17.5%) patients, including diarrhea, nausea, vomiting, chest pain and an increase in liver transaminase levels. The adverse drug reactions were mild to moderate and caused early discontinuation of the study drug in 2/40 (5.0%)	N/A
Cai Q, et al.	Total numbers of adverse reactions were 4 in favipiravir 5.71% reported diarrhea, respectively. None of reported for vomiting, nausa, and rash.	Total numbers of adverse reactions were 24 in LPV/RTV groups. 11.11% reported diarrhea, respectively. 11.1, 13.33, 8.89% of LPV/RTV group reported vomiting, nausa, and rash, respectively.
Doi Y, et al. (Japan)	Among 82 patients, total 144 adverse events. The most common was hyperuricemia (84.1%). Of 32 patients who had serum uric acid level determined, 24 had the level normalized to below 7 mg/dL, with the highest being 8.8 mg/dL. Serum triglyceride elevation (11.0%) Serum alanine aminotransferase elevation (8.5%).	N/A

*LPV/RTV* lopinavir/ritonavir, *N/A* not avialable

**Fig. 6:**
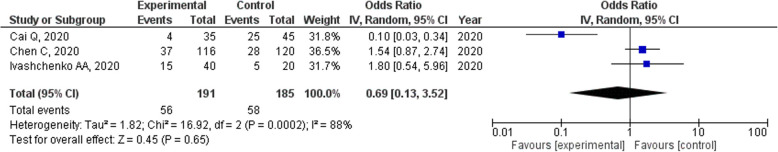
Forrest plots of the number of adverse events for patients with COVID-19 who were treated with favipiravir

Concerning frequent adverse drug reactions, diarrhea or digestive tract reactions were reported in three studies, decreased albumin levels were reported in one study, and hyperuricemia was reported in one study. Serum uric acid levels were also determined in two studies. The number of adverse drug reactions was not significantly different among three trials (OR = 0.69, 95% CI = 0.13–3.52, *p* = 0.62; Fig. 6).

## Discussion

The present systematic review and meta-analysis using the limited available evidence revealed that favipiravir has high promise for treating patients with COVID-19. Among patients with moderate COVID-19, favipiravir accelerated viral clearance after 7 days of treatment. Favipiravir also contributed to clinical improvement, especially after 14 days of treatment. Drugs other than antiviral agents, such as nafamostat or methylprednisolone, can be used in combination with favipiravir for patients with moderate or severe COVID-19. However, we must await well-designed studies assessing effectiveness of favipiravir in patients with COVID-19, including examinations of the different doses and durations of therapy in patients with different levels of disease severity.

Favipiravir, which has displayed efficacy against many RNA viruses, acts by inhibiting RNA-dependent RNA polymerase, and it is one of several potential drugs that may be repurposed for treating COVID-19 [4, 7, 27]. In this study, although different comparators were used among the studies, the meta-analysis estimated that favipiravir was associated with a significantly higher likelihood of viral clearance on day 7. Contrarily, the proportions of viral clearance were not significantly different by day 14. The viral load of SARS-CoV-2 peaks around symptom onset or a few days thereafter, and the virus becomes undetectable within approximately 2 weeks [28]. In addition, among most eligible studies, the duration of favipiravir therapy was 14 days. The lack of a significant difference in the proportion of viral clearance at day 14 between the treatments may reflect the natural course of viral shedding. However, the emergence of patients and healthy viral carriers with early viral RNA clearance is a major concern for disease management and infection control measures. In fact, the study found that the median time to viral clearance among patients who were administered favipiravir on day 1 after onset was less than 12.8 days, compared with 17.8 days for patients who started treatment on day 6 [17]. Favipiravir is an oral drug; therefore, it is easy to administer to patients with asymptomatic or mild COVID-19. This result strengthened the importance of early favipiravir administration as well as the role of the drug in the management of early-stage or asymptomatic COVID-19.

Favipiravir contributed to significant clinical improvement by 14 days, but not 7 days, after treatment initiation. Contrarily, the time to body temperature normalization was approximately 2 days [18, 19]. The definition of clinical improvement differed among the studies; however, variables that defined clinical improvement included the respiration rate, oxygen saturation, cough relief, and chest CT improvement. These clinical signs and symptoms were affected by lung injury or pneumonia. Under the various clinical manifestations of COVID-19, ranging from an asymptomatic disease course to the clinical symptoms of acute respiratory distress syndrome and severe pneumonia, the lungs, which have extremely slow cell turnover, are the primary organs affected by SARS-CoV-2 [29]. Most patients in the present study had mild-to-moderate COVID-19, and favipiravir treatment may have contributed to lung recovery within 14 days from the initiation of treatment. A study of patients with asymptomatic or mild COVID-19 comparing early and late favipiravir initiation revealed a significant difference in the duration of hospitalization [19]. The result demonstrated the necessity of early favipiravir initiation even for patients with asymptomatic or mild COVID-19 before pneumonia develops or lung damage worsens [30]. In addition, the standard dosage of favipiravir for influenza was 1600 mg twice daily on the first day followed by 600 mg twice daily for a total of 5 days [5]. Most of the eligible studies followed the standard regimen, and the treatment duration was generally 14 days. However, some studies increased the dose to 1800 mg twice daily on the first day followed by 800 mg twice daily [28]. The losing variations are likely attributable to the lower favipiravir EC_50_ described against influenza than against Ebola and SARS-CoV-2 [31, 32]. Various dosing regimens have been proposed based on the type of infection indication [33]; a loading dose of 2400–3000 mg every 12 h (two doses) has been considered for the treatment of COVID-19, followed by a maintenance dose of 1200–1800 mg every 12 h [31, 32]. Rattanaumpawan et al. examined the loading dose of favipiravir and concluded that a low loading dose (≤45 mg/kg/day) was a poor prognosis factor for early clinical improvement. Doses at the higher end of the dosing range should be considered for the optimal treatment of COVID-19.

A review article demonstrated that favipiravir had a tolerable safety profile in terms of total and serious adverse effects compared with other drugs used for short-term treatment [33]. This is the compatible with the present systematic review and hyperuricemia was observed in 84.1% of patients with asymptomatic or mild COVID-19 patients in one study [19]. Although there is limited clinical experience with favipiravir for COVID-19 treatment, the present study demonstrated that serious adverse events induced by favipiravir were not observed. In addition, due to a risk of teratogenicity and embryotoxicity, the Ministry of Health, Labor and Welfare in Japan has therefore only granted conditional marketing approval for its production and clinical use for influenza virus infection [7]. This safety information will play a critical role of favipiravir in COVID-19 patients, especially pregnant women.

The present study had some limitations. First, only some of the studies included a comparator arm. In addition, although favipiravir treatment in most studies was followed by standard care for influenza, the dose and duration were not same among the trials. However, we included the studies that had no comparator arm and different dose and duration into the assessments for the proportions of viral clearance and clinical improvement if the outcome variable were presented. Second, the observation points of the primary outcomes were not strictly 7 and 14 days after treatment initiation in all studies. Third, the definition of clinical improvement differed among the studies. Despite these limitations, in an effort to expand the role of favipiravir in the clinical management of COVID-19, especially for patients with asymptomatic and mild-to-moderate disease, it was crucial to quickly examine the efficacy and safety of favipiravir.

## Conclusions

Our study revealed that favipiravir can promote viral clearance within 7 days and clinical improvement within 14 days, especially in patients with mild-to-moderate COVID-19. The early initiation of treatment with favipiravir can contribute to positive outcomes for COVID-19. This systematic review and meta-analysis demonstrated the high potentiality for the use of favipiravir for COVID-19. In particular, since favipiravir is the oral form, it is easy to administer for asymptomatic or mildly ill patients with COVID-19. However, there is an urgent need for additional evidence, especially trials assessing different doses and durations of therapy and patients with different levels of disease severity.

## Supplementary Information

**Additional file 1.**

## Data Availability

All relevant data are included within the paper and the Supporting Information file.

## Abbreviations

COVID-19: Novel coronavirus disease
SARS-CoV-2: Severe acute respiratory syndrome coronavirus
RdRp: RNA-dependent RNA polymerase
RCT: Randomized clinical trials
SOC: Standard of care
CT: Computer tomography
LPV/RTV: Lopinavir/ritonavir
HQ: Hydroxychloroquine
AZ: Azithromycin
SD: Standard deviation
IQR: Interquartile range
NA: Not available
OR: Odds ratio
HR: Hazard ratio
DIC: Disseminated intravascular coagulation
ICU: Intensive care unit
SpO_2_: Blood oxygen saturation
MV: Mechanical ventilation
ECMO: Extracorporeal membrane oxygenation
NEWS2: National Early Warning Score
RBC: Red blood cell
