# Supplementary material for: Favipiravir for the treatment of patients with COVID-19: a systematic review and meta-analysis
Source: BMC Infect Dis. 2021 May 27;21:489. doi: 10.1186/s12879-021-06164-x (PMC8159019; doi:10.1186/s12879-021-06164-x)
Supplement: Supplementary file 1 — Additional file 1. [file 12879_2021_6164_MOESM1_ESM.docx]

**Supplement**

**S1 Fig. 1.**


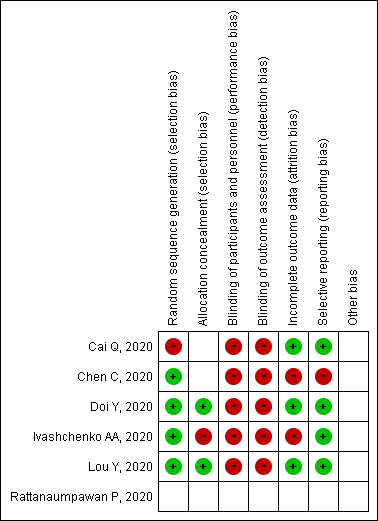


**S2 Fig. 2.**


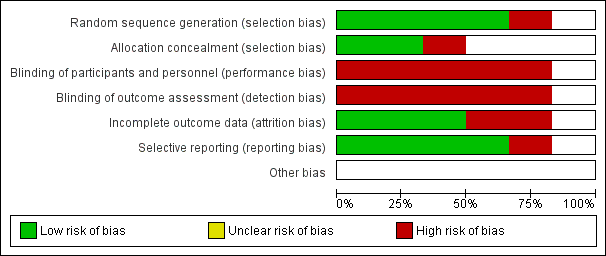


**S3 Fig. S3**

Proportion of Effect for viral clearance among comparators of favipiravir **a. by day 7** and **b. by day 14** from the initiation of treatment

**a.**


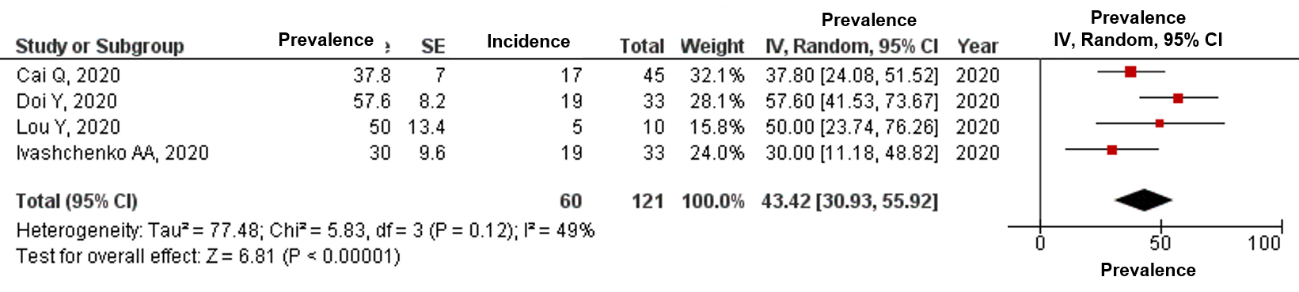


**b.**

**
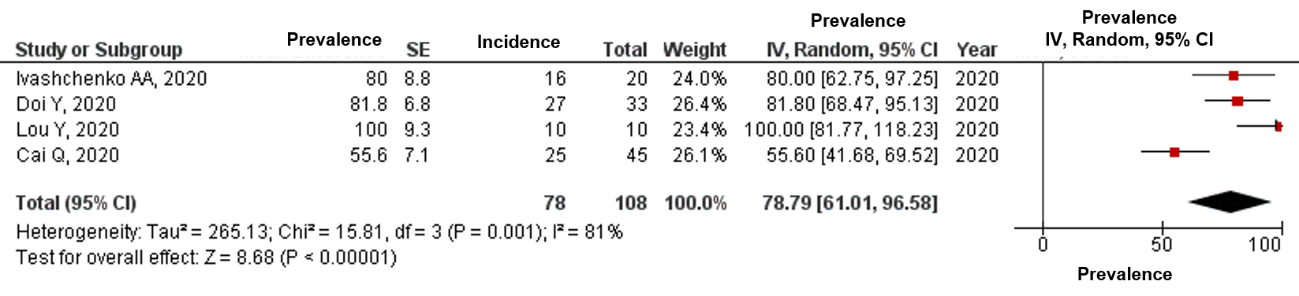
**

**S4 Fig. S4**

Proportion of Effect for clinical improvement among comparators of favipiravir **a. by day 7** and **b. by day 14** from the initiation of treatment

**a.**

**
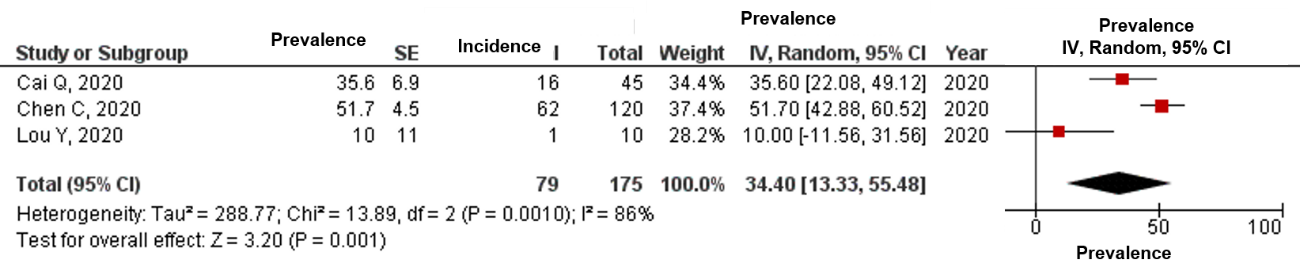
**

**b.**

**
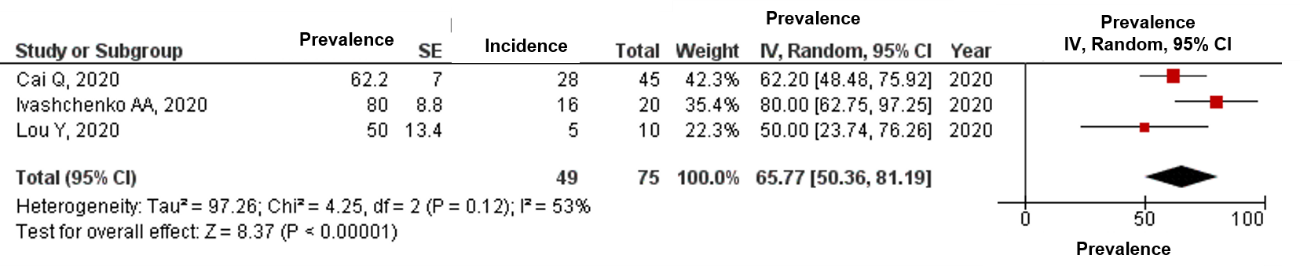
**
